# The Importance of the Hedgehog Signaling Pathway in Tumorigenesis of Spinal and Cranial Chordoma

**DOI:** 10.3390/jcm8020248

**Published:** 2019-02-15

**Authors:** Reza Akhavan-Sigari, Walter Schulz-Schaeffer, Amanda Angelika Harcej, Veit Rohde

**Affiliations:** 1 Department of Neurosurgery, University Medical Center Göttingen, Georg-August-University Göttingen, 37075 Göttingen, Germany; amanda.harcej@gmx.de (A.A.H.); veitrohde@gmail.com (V.R.); 2 Department of Neuropathology, Saarland University Medical Center and Saarland University Faculty of Medicine, 66421 Homburg, Germany; Walter.Schulz-Schaeffer@uks.eu

**Keywords:** hedgehog signaling cascade, tumorigenesis, spinal and cranial chordomas

## Abstract

Chordomas is rare malignant bone tumors thought to arise from remnants of embryonic notochord along the spine, frequently at the skull base and sacrum. Although chordoma is slow growing tumors, while are extremely recurrent, and aggressive, as well as the rate of prognosis remains poorly. Radical surgery and high-dose radiation are the most used treatments. Currently, there is no effective chemotherapeutic standard for chordomas. The Hedgehog (HH) pathway adjusts various processes included in expansion and differentiation of tissues and organs throughout the fetus’s life, furthermore cell growth and differentiation in the adult organism, of the cell in an adult organism, in which acute anesthesia is involved in multiple cancers. To study the role of signaling the hedgehog in the base of the skull and sacrum chordomas, the expression of SHH and GLI-1 levels were detected immuno histochemically, Additionally, PTCH-1 and GLI-1 expressions were distinguished by in- Situ- hybridization. Based on the findings presented herein, it is likely that the HH signal cascade was revealed even in cranial, where consecoently spinal chordoma and their recurrences play an important role. Our staining exhibited a canonical, ligand- dependent and autocrine Hedgehog signaling in skull base and sacrum chordomas including relapse. Due to the high levels of SHH and GLI-1 expression in all investigated chordoma samples, the study suggests a possible autocrine ligand-dependent activation of the canonical HH signaling cascade. A paracrine or non-canonical pathway cannot be excluded. Our results suggest that Hedgehog-inhibitors, like SHH-, GLI- and SMO- inhibitors, might serve as a potential and effective target for the treatment of chordomas.

## 1. Introduction

Chordoma is a rare, slow-growing type of bone neoplasm arising from cartilage cells [1]. It is predominant in male with onset usually seen at around the sixth decade of life, and have an approximate incidence of 0.1–0.8 per 1,000,000 individuals per year [2,3]. Chordoma is particularly prevalent in the skull base region and in the area of the sacrum [3,4]

Although it is a slow-growing tumor, but is a locally invasive and destructive. Chordomas are considered to be complicated tumors to treat. In patients with locally advanced neoplasm, most of them experience death within 12 months of disease progression [5,6]. Therapeutic strategy at the present time consists of radical surgical resection with subsequent irradiation. En bloc resection with negative margins remains the treatment of choice. However, the magnitude of its extension makes radical surgery difficult in up to 50% of the cases [7]. Moreover, the recurrence rate of these tumors is markedly high [3,8]; Therefore, the treatment of chordomas is still a challenge.

Ample evidence indicates a strong genetic association of chordomas with the notochord. The “T” gene, located on chromosome 6q27, encodes the “Brachyury” protein and is evolutionarily favorable, where plays a critical role in the development of the notochord and in defining the midline of bilaterian organisms [9].

Conventional and molecular cytogenetic analyses revealed chromosomal gains of 7q and losses of 1p and 3p to be the most prominent alterations in chordoma [10]. In addition, loss of heterozygosity (LOH) and genome-wide linkage studies have already been successfully used to narrow down and define candidate regions for chordoma development on 1p36.13 and 7q33. [11]

However, many specific genes or altered transcripts have yet to be determined. Using comparative genomic hybridization (CGH) in chordomas, the most frequently lost regions were in 3p and 1p, while gained genomic regions mostly mapped to chromosomes 7 and 20 [12].

Recent studies have demonstrated that chordoma formation occurs in the third embryonic week from the notochord (Chorda dorsalis) [9]. In addition, notochord secrets various signaling molecules during this early embryogenesis, including the Sonic-Hedgehog (SHH) molecule for regulating proliferation, differentiation and survival of embryonic cells [13,14,15,16]. Previously, numerous studies demonstrated the role of the hedgehog signaling cascade in tumor growth, and metastasis in a variety of human tumors such as adamantinous craniopharyngioma, glioblastoma, medulloblastoma and rhabdomyosarcoma [17,18].

The involvement of an extracellular signaling pathway such as the hedgehog signaling pathway in the development of chordoma and its recurrences remains unclear. There is currently no chemotherapeutic recommendation for the treatment of chordomas and their relapses, no drug has been revealed to be effective in locally advanced and metastatic chordomas [8]. If a signaling cascade is detected, a therapeutic approach in term of chemotherapeutic agent might be conceivable.

Hedgehog Proteins (HH) are originally discovered in Drosophila, along with multitude else ingredients of their signal transmission devices [19].

Drosophila has been described to have an isolated HH gene, while vertebrate HH signal transduction has been indicated to be involved in three HH homologues with the various spatial and provisional dispensation template including Indian (IHH), Sonic (SHH), and Desert Hedgehog (DHH) [16], of which Sonic has been well investigated

It is noteworthy that a receptor called Patched (PTCH) is known for the HH signaling pathway, which is divided into PTCH1 and PTCH2 [20,21]. In humans, a warning signal hydraulic cascade in the target cell begins with ligand binding of HH ligand to the Patched 1 protein (PTCH), as a 12-span transmembrane protein. In the absence of the HH ligand, the PATCH catalytically interdicts the actuality of a protein similar to the seven receptors of the SMO protein [22,23]. It has been also suggested that a small intracellular molecule act as an agonist of the SMO and can be transmitted outside the cell by PATCH, barricade it’s binding to the SMO. The HH connection to the PITCH findings in the absence of PATCH stir and the activation of the SMO are involved in transmitting the HH warning to the cytoplasm [24,25]. The HH signal is transmitted via an alteration of the balance between the activator and repressor forms of the Ci (cubitus interruptus)/GLI family of zinc-finger transcription factors. The GLI transcription factors, GLI1, GLI2, and GLI3, are known to be effectors of Hedgehog signaling, where GLI 1 and GLI 2 act as transcriptional activators and GLI 3 as a transcriptional repressor [25].

The expression of GLI 1 is strongly dependent on the activation of the HH warning and is therefore used as reading path activation. In the absence of the HH ligand, SMO activities can be blocked with PTCH and complete GLI proteins are processed in a proteolytic manner to produce a GLI repressor, mainly isolated from GLI 3 that suppresses the target genes of HH. An increasing body of evidence suggests that GLI1 and GLI2 are involved in activating of the target gene, while they are also capable of inactivating the negative regulator, PTCH1. GLI is considered to be a repressor of the SHH target genes [26,27]. Since GLI1 stimulates PTCH transcription, therefore both GLI1 and PTCH1 can be regarded as target genes, as well as reliable markers for an active HH/PTCH signaling cascade [20]. It has already been proven that dysfunctions of the SHH signaling cascade lead to the development of various diseases or neoplasia [18]. Ptch1 is a tumor suppressor gene, whereas SMO is a proto-oncogene [28,29].

On the other hand, there are also non-canonical mechanisms that activate GLI1 transcription. However, many recent studies have demonstrated that HH signaling cannot be processed through GLI activation, but can act via other signaling pathways, such as the RAS (Rat Sarcoma) and Transforming Growth Factor Beta (TGFb) signaling cascade [30,31]. In the current study, we investigated whether SHH signal cascade play a role in the formation of chordomas and their recurrences. Since there is currently no standardized chemotherapeutic recommendation for the treatment of chordoma and its recurrences. However, a chemotherapeutic approach for the treatment of these patients may be conceivable if evidence of an active SHH signaling cascade is available.

## 2. Experimental Section

### 2.1. Patient Collective

In the current study, we assessed 33 paraffin-embedded chordoma tumor samples (snap-frozen tissue samples were also available for 7 sample) obtained from 26 patients (8 male, 18 female; median age: 66 years), 6 fresh-frozen, conventional chondrosarcomas (6 patients; 4 male, 2 female; median age at diagnosis: 54 years; 1 clivus, 3 femur, 2 pelvis; 3 grade 1, 3 grade 2) and pooled material of short-term cultures of 2 vertebral discs (both male; age 47 and 63 years) from the files of the Institute of Pathology, University

Fifty-six patients were initially enrolled in the present study from 1982 to 2017. Only those patients with the diagnosis of conventional chordoma were included in this study. Out of 56 patients, 20 patients were finally included in the study by using electronic files, where electronic desk studies were available. The treatment of patients was performed in the University Medical Center Göttingen, Germany. Among enrolled patients, 12 patients (60%) suffered from conventional cranial and 8 patients (40%, conventional chordoma) had spinal chordoma.

The average age of patients with cranial chordoma at the time of diagnosis was found to be 49 years, the youngest patient being 10 and the eldest ones 89 years old. The average age of patients with spinal chordoma was recorded as 57, which youngest patient being 18 and the oldest patient 80 years old (Table 1).

Useful tumor material for the immunohistochemistry and in situ hybridization was available for 14 (70%) patients. Patients were divided into 2 groups: 8 (57%) cranial chordomas and 6 (43%) spinal chordomas.

### 2.2. Immunohistochemistry

Samples were intraoperatively collected and routinely dehydrated in a series of fresh alcohol solutions with increasing concentrations (one bath of 50%, 70%, 80%, 96% methanol, and four baths of 100%, methanol) for 1 h. Afterward, samples were cleared in xylene (three baths, 1 h each), and subsequently embedded in paraffin. Immunohistochemistry was performed on all samples of cranial and spinal chordomas with the following antibodies: anti-SHH (1:500, Rabbit Polyclonal Antibody, Dunn, Asbach, Germany), and anti-Gli1 (1:100 Rabbit Polyclonal Antibody, Dunn, Asbach, Germany). The reference antibodies used are cited in literature for IHC. All slides were simultaneously processed under identical condition using standard methods. The tissue sections were pretreated for antigen retrieval with citrate pH 6.0 in the microwave at 80 °C. Afterward, these tissues were remedied with an initial antibody and then stained using a combination of Avidine-Biotin Peroxidase (Immunotech, Marseille, France) or an Alkaline Phosphatase Detection Kit (Vector, Burlingame CA, USA) according to standard immunohistochemical methods [32]. All slides were run together in the same situations, where negative control slides were considered. Positive and negative control parts were included for all antibody and slide pretreatment, respectively. In incubation, the tissue microcymide slides (TMA) was removed with the early antibody, as a negative control for any antigen retrieval regimen. Using the membranous and/or coarse cytoplasmic staining, TMAs were separately appraised by the following criteria for a particular staining. To avoid nonspecific binding, the tissue samples were blocked for 3 min in H_2_O_2_/TBS (Tris Buffered Saline) 3%, followed by incubation in TBS/0.1% Triton X-100 for 10 min and in 0.2% casein for 20 min with. The primary antibody was applied to the samples for 90 min at room temperature and rinsed with TBS/0.1% Triton X-100. After that, the secondary antibody was added to the samples for 30 min at room temperature.

On the other hand, it is noteworthy that paraffin sections were incubated with or without abundant antigen peptide along with antibodies for successful blocking of the IHC signal. A negative control without primary antibody can be very useful for IHC. In addition, a better negative tissue, that is not expressed the protein of interest, can be effectively valuable. This type of antibody validation is not only considered to be an appropriate strategy, but also is capable of providing reliable results for IHC (Immunohistochemistry).

### 2.3. Immunohistochemical Scoring for Shh and Gli1

Immunoreactivity was investigated by two pathologists who were blind to all clinical information and other histological detections. Immunoreactivity for the proteases was detected as characterized previously [33]. Every tumor was detected according to the severity of nucleic or cytoplasmic staining (no staining = 0, fragile staining = 1, temperate staining = 2, strong staining = 3) and stained cells (0 = none, 1 = less) from 25%, 2 = 25–50%, and 3 = more than 50%). The two parameters were considered to measure GLI1 and SHH expression levels (between 0 and 6). The cells were scored as negative expression (score 0), weak expression (1), moderate expression (score 2), strong expression (3–6 scores).

All slides were simultaneously run under identical conditions and negative control slides were included. Positive and negative control sections were included for each antibody and pre-treatment of slides, respectively.

The negative controls were considered for each antigen retrieval regimen, where the primary antibody was omitted from the incubation step.

### 2.4. In Situ Hybridization

Sensors and protein RNA diogoxyginic marker were manufactures by a DIG RNA chain kit based on the manufacturer’s instructions (Boehringer Mannheim). All sections, both positive and negative controls, were processed under identical conditions.

The negative controls sections were incubated with the PTCH1 Sense and GLI1 Sense probes. The in situ hybridization was conducted as explained previously [34,35]. In summary, water disinfection sections were remedied with Proteinase K (20 μg/mL) for 20 min and then dissolved in 4% paraffinic acid by PBS for 5 min. Moreover, all paraffin sections were incubated for two h at 57 °C in the hybridization oven, deparaffinized twice in xylene for 20 min, followed by immersion in a descending ethanol series (96%, 96%, 75%, 50%, 30%, DMPC (Dimyristoylphosphatidylcholine)-H_2_O) (DMPC, Dimyristoylphosphatidylcholine) for three minutes. Samples were washed with 50% wax in 2 × SSC at 37 °C for 2 h and then hybridized at 42 °C overnight with 5 μg/mL diphoxidine-labeled probe. The reactions were treated with 4 × SSC, 2 × SSC, 20 μg/mL RNase A, 1 × SSC, and 0.1 × SSC at 37 °C, 15 × 3, then eluted in in buffer 1 (100 mM/L Tris-Cl, 150 mol/liter NaCl, pH 7.5). Moreover, these samples were incubated for 30 min in a buffer of -2% goat serums, 0.1% TritonX-100 in buffer 1, at room temperature for one hour at room temperature for one hour at room temperature for one hour. Afterward, samples were incubated with anti-bacoxixinin alkaline phosphatase (Roche) solution at room temperature for one hour at a dilution of 1:500, followed by incubation in buffer blocking. Samples were washed by buffer 2 (100 milliliters per liter Tris-Cl, 100 milloles per liter NaCl, 50 milloles per liter MgCl_2_, pH 7.5), and 3 (100 milloles per liter Tris-Cl, 100 milloles per liter NaCl, 50 mM/L MgCl_2_, pH 9.5), 2 × 10 min. The color response was performed with NBT/BCIP solution (Roche); solutions at a dilution of 1:500 including 10 mmol/L levamisole in the dim, in which blue staining revaled powerful hybridization. Probe sensors were detected in the whole hybridization as negative controls and no positive signals. Finally, the tissue sections were covered with an aqueous mounting medium (Aquatex®, Sigma-Aldrich, Temecula, CA, USA) for preservation.

### 2.5. Ethics Approval and Consent to Participate

All procedures performed in studies involving human participants were in accordance with the ethical standards of the institutional and/or national research committee and with the 1964 Helsinki Declaration and its later amendments or comparable ethical standards. The study protocol was approved by the Ethics Committee of University Medical Center Göttingen, Germany.

## 3. Results

### 3.1. Immunohistochemistry

Overall, 14 (100%) cranial chordomas from eight patients were immunohistochemically assayed for expression levels of Shh and GLI1. All enrolled tumor samples (14 samples) were divided into seven primary tumors, including first recurrences (five samples) and second recurrences (two samples). Based on the data presented herein, 10 specimens (71%), including all seven primary tumors and three first relapses from seven (87.5%) patients, were found to be Shh positive (+) and Gli1 positive (+). The remaining four (29%) cranial samples from two (25%) patients, belonged to two first and two second relapses, which were reasonably positive for GLI1. Overall, GLI1 with 14 (100%) positive results was slightly more responsive to all eight (100%) patients, when comparing with Shh positive samples (10 samples; 71%) from seven (88%) patients (Table 2). In addition, the expression rate of Shh and GLI 1 raised by the first recurrence, while their expression levels exhibited a decreasing trend in the second recurrence; however, a final interpretation of this event cannot be done because different factors may be involved in this condition, such as the sample size that should be considered in the light of a limitation.

In addition, 18 (100%) spinal chordomas, including twelve primary tumors and six first relapses, from six patients were immunohistochemically examined for the expression levels of Shh and GLI1. There were 17 (94%) samples, more precisely 11 primary tumors and six first recurrences, of which all six patients exhibited both Shh expression (+) and GLI1 expression (+). A spinal sample (6%) from one (17%) patient showed a positive reaction for Shh, which was recorded as a primary tumor. Moreover, among all six patients, 18 samples revealed Shh expression (+) as compared to 17 samples with GLI1 expression (+), (Table 3). Based on the data presented in Table 3, our findings exhibited that GLI1 and Shh levels were markedly increased in score 3, when comparing with other scores.

**Figure 1:**
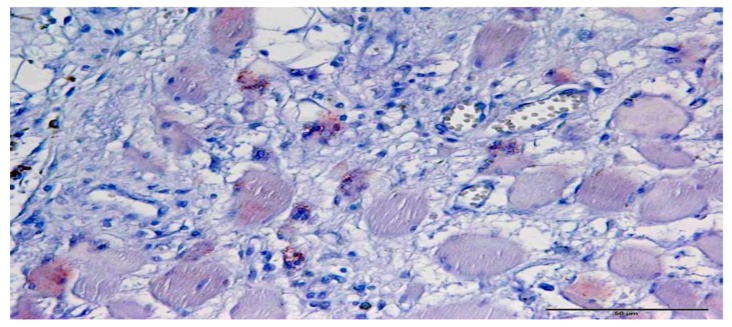
Immunohistochemical staining of Gli1 expression in a spinal chordoma. The expression strength of the cytoplasmic granular staining of the tumor cells corresponds to the score 1 (magnification 400×).

**Figure 2:**
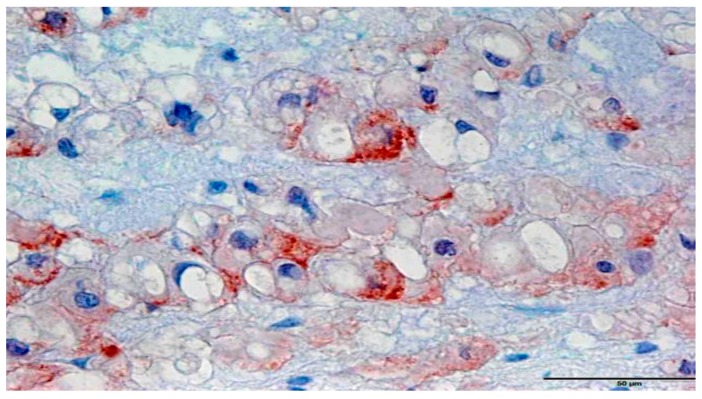
Immunohistochemical staining of Gli1 expression in a cranial chordoma. The expression strength of the cytoplasmic granular staining of the tumor cells corresponds to the score 3 (magnification 400×).

In summary, all 19 cranial and spinal primary tumors of 14 patients showed expression of Shh and GLI1. In addition, out of 14 patients, 11 cranial and spinal tissues with first relapses, as well as the two cranial samples with second relapses exhibited Shh and/or GLI1 expression levels. (Figure 1 and Figure 2).

### 3.2. In Situ Hybridization

A total of 14 cranial chordomas were subdivided into seven primary tumors; five first relapses and two second recurrences that belonged to eight (100%) patients. Cranial chordomas were examined for the expression of Ptch1 and GLI1 by in situ hybridization. Four (57%) primary cranial tumors from three (38%) patients revealed a positive response, of which two (29%) primary tumors were positive for Ptch1 (+) and two (29%) for GLI1 (+). Nevertheless, no response to the PTCH1 and GLI1 markers was found for the first and second cranial recurrences (Table 4).

Furthermore, 18 spinal cord chordomas from six patients (100%) were subdivided into twelve primary tumors and six first relapses, were tested for expression of Ptch1 and Gli1 by in situ hybridization. Of these, eight (67%) primary tumors from three (50%) patients exhibited a positive response, three (25%) samples from two (33%) patients were PTCH1 positive (+) and five (42%) samples from three (50%) patients were found to be positive for GLI1 (+). On the other hand, negative responses for PTCH1 and GLI1 were detected in the first spinal chordoma recurrences (Table 4).

**Figure 3:**
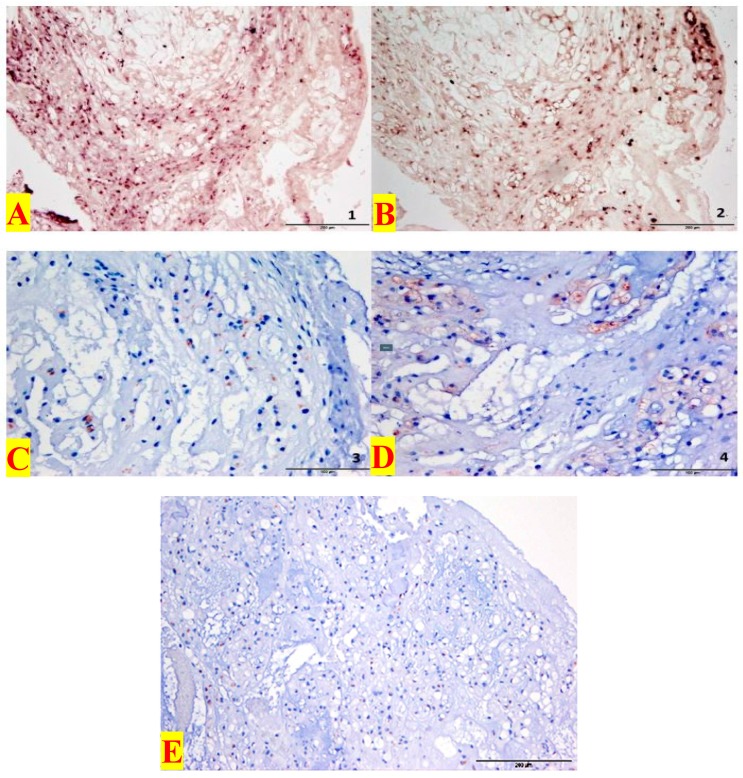
Comparison of a cranial chordoma sample from a patient after positive in situ hybridization (Ptch1 (+), Gli1 (+)) and immunohistochemical staining (Shh (+) and Gli1 (+)) in comparison: (**A**): In situ hybridization Ptch1 (+) at 100× magnification. (**B**): In situ hybridization Gli1 (+) at 100× magnification. (**C**): Immunohistochemistry Shh (+), score 2, magnified 200 times. (**D**): Immunohistochemistry Gli1 (+), Score 2, in 200× magnification. (**E**): For negative control, the primary antibody was substituted by PBS (Phosphate-buffered saline). All slides were run simultaneously under identical conditions and negative control slides were included. Slides in which incubation with primary antibody was omitted served as the negative controls for each antigen retrieval regimen.

Furthermore, the expression of PTCH1 and GLI1 was only visible in the tumor cell nucleus in cranial, spinal primary tumors and relapses, (Figure 3). The neighboring stromal cells showed no positive expression for PTCH1 and GLI1.

## 4. Discussion

Chordoma patients experience death within 12 months of disease progression [2,5,6]. The treatment of choice in the treatment of chordomas is currently surgical resection followed by radiation [8]. Classical radiotherapy with high-energy photons offers sufferers a short-term improvement in the clinical picture [36]. Due to the rarity of the tumors, there is no evidence-based study for accompanying chemotherapy in the treatment of chordomas and their recurrences [8].

In a recent study, molecular cytogenetics demonstrated that gains of chromosomal material in chordoma were clearly high at 7q (42%), 12q (21%), 17q (21%), 20q (27%) and 22q (21%). DNA sequence losses occurred most frequently at 1p (21%), 3p (36%), 4q (27%), 10q (21%) and 13q (24%). In summary, these tumors are characterized by non-random genomic copy number alterations, where losses are more frequent than gains [37].

The notochord expresses various signal molecules, including the sonic hedgehog molecule for regulation of the proliferation, differentiation, and survival of embryonic cells [13,14,15,16]. The Sonic-Hedgehog molecule belongs to the HH proteins, which are extracellular signaling molecules and play a key role in the embryonic development of many organisms [38]. Previously, active Hh signaling has been revealed to be associated with both tumor growth and metastasis in many kinds of malignancies such as basal cell, ovarian cancer, prostate cancer, breast carcinomas, clear cell renal cell and lung carcinoma, as well as glioblastoma, medulloblastoma, Rhab-domyosarcoma and adamantinösen craniopharyngeoma [14,39,40,41,42]. The question arose as to whether intracellular signaling passages, like the HH signaling pathway, are also involved in the development of chordomas and their relapses. In addition to surgical resection and radiation, the use of Hh inhibitor in combination with chemotherapy may be conceivable for detection of a signal cascade. For this purpose, the expression of Shh and GLI1 were immunohistochemically evaluated on formalin-fixed paraffin-embedded tissues from 14 patients including 14 cranial and 18 spinal tumor blocks. Furthermore, these samples were assessed for PTCH1 and GLI1 expression levels by in situ hybridization to detect an active Shh signal cascade. Pathological activation of the Shh signaling cascade, caused by overexpression, mutation or loss of function of the signaling molecules, can lead to the development of neoplasias. Teglund and Toftgård discussed three different signal transduction possibilities for the HH cascade in tumorigenesis.

The first (non-canonical) model describes the intrinsic, ligand-independent SHH signaling activation by mutations that result in loss of functions or gain of functions and thus increasing activity [43,44]. For instance, a lower expression of PTCH may indicate a possible PTCH mutation or a PTCH loss-of-function, where has already been investigated in basal cell carcinoma, Gorlin-Goltz syndrome, medulloblastoma, and rhabdomyosarcoma [24,40,45,46]. In this case, PTCH can no longer inhibit Smo, leading to activation of the signal cascade [21,28,42].

The second canonical type is autocrine and Shh –dependent, where is assumed to involved in an autonomous HH ligand production by the tumor cells [43,44]. This mechanism has been discovered in various malignancies including glioblastoma, melanoma, breast, prostate, colon, pancreatic, and small cell lung carcinoma [47,48,49,50,51,52].

Thirdly, in paracrine canonical model, the tumor cells express the HH ligands by which activate the HH signaling pathway in neighboring stromal cells [44]. These stimulate the tumor cells through a paracrine feedback mechanism, such as the production of angiogenesis factors (IGF, VEGF), interleukin 6, and the Wnt signaling pathway for further ligand production [21]. Previous reports indicated a predominantly paracrine pathway in multiple myeloma and lymphoma [43,53]. It is worth noting that both autocrine and paracrine pathways are involved in pulmonary, pancreatic, esophageal, colon and prostate cancers [21]. In brief, tumor progression is promoted by all three processes [31,42].

Based on the data presented herein, all 19 investigated primary tumors (cranial and spinal) exhibited positive response for Shh (+), while 18 samples (95%) were positive for Gli1 (+). Of the 11 first recurrence samples (cranial and spinal), 9 (81%) samples were also found as Shh positive (+), while Gli1 positive expression (+) was found in all samples. Due to the strong expression of Shh and Gli1 in the tumor cells, canonical activation of Shh pathway is likely to be possible in cranial and spinal chordomas and its recurrence.

The results of the in situ hybridization in our study were partially positive for PTCH1 and GLI1 in primary conventional cranial and spinal chordoma. In contrast to positive expression of PTCH1 and GLI1 in our findings, it is essential to show that tumor cell RNA is preserved and available for hybridization. Tumor cell RNA may alternatively be focal and weak, contributing to false-negative interpretation. False-negative results may be a consequence of RNA degradation and are more frequent in decalcified tissues [54].

In contrast, Scheil et al., [10] could not demonstrate that SHH gene were overexpressed in chordoma. None of the genes involved in the SHH pathway was transcriptionally activated in chordoma or chondrosarcoma. However, the gene coding for osteopontin (SSP), which has been shown to be transcription­ally activated by GLI1, was upregulated in four chordomas and one chondrosarcoma [10]. Other than our study, a differentiation of chordoma types has not been notified in this study. This might explain the different results of SHH expression in the study of Scheil et al. as compared with ours.

A question raised whether an autocrine/paracrine regulatory mechanism is responsible for this condition, while cannot be completely deduced. In a potential paracrine activation, Shh expression in the tumor cells can be associated with the increased interpretation of Ptch1 and Gli1 in the adjacent stromal cells [38], which was not evident in this series of experiments. Based on the date presented herein, the expression levels of GLI1 and PTCH1were observed only in the nucleus of the tumor cells during in situ hybridization, but no expression was found in the surrounding stromal cells.

A possible functional role for the autocrine activation has been found based on current evidence. An intrinsic, ligand-independent SHH signaling was not expected due to the high levels of Shh expression presented in the primary tumors and first recurrence [42]. Since Gli1 was also positive in all investigated samples, where Shh seems to activate the cascade as a ligand and the transcription of the Gli1. Furthermore, to exclude a possible PTCH dysfunction by targeted DNA sequencing, the chordoma samples needed further investigated for known mutations [54]. Thus, an unknown process of the HH signaling cascade cannot be ruled out in chordomas, where is possibly active and may be capable of interacting with the classical Shh pathway [10,27,55].

Previously, the well- known involvement of the HH signaling cascade in tumorigenesis have had a special priority, as well as preparation of substances, which can therapeutically inhibit this signaling pathway.

For an effective blockade, several key positions in the active process are conceivable. The first possibility is to suppress the binding of the Shh ligand to its receptor, Ptch1. In addition to the HH antibody 5E1, roboticininin, a synthetically produced molecule is known to have an inhibitory effect on the secondary signaling cascade through extracellular binding to Shh [56,57]. It is also likely to inhibit the function of the Shh ligand and thus prevent binding to the receptor Ptch1 [58].

Another possibility is the inhibition of the SMO protein [59]. So-called SMO-inhibitors, such as cyclopamine or vismodegib, are currently being used as monotherapy due to promising preclinical results in clinical trials to prevent medulloblastoma and basal cell carcinoma [21,60,61]. The last option is inhibition of Gli’s activity. This can be indirectly done via inhibition of SMO transport (HPI-4: Ciliobrevin A) and directly by binding interference of GLI to the promoter (GANT 58, GANT 61) [30,56,59,60,61,62,63,64].

Due to the high expression of Shh and GLI1 in all investigated chordoma samples, the present results suggest a ligand-dependent SHH signaling cascade activation in cranial and spinal chordomas, as well as their relapse. Suppression of the binding of the ligand Shh to PTCH1 by 5E1, robotnikinin or RU-SKI 43 could potentially stop tumor progression in our investigated chordomas. Blotta et al. suggested that the simultaneous use of GLI- and SMO-inhibitors serve as a more potent combination therapy in multiple myeloma for inhibiting the HH signaling cascade at multiple interfaces [43]. Based on our findings, the use of GLI inhibitors such as GANT 58, GANT 61, and HPI-1-4, which act directly on the target protein, could potentially prevent further tumor growth of the chordomas. Indirect inhibition of GLI by SMO inhibitors may also disrupt the active HH signaling cascade and should be further investigated [10,31,53,64,65].

## 5. Conclusions

Low number of samples does not allow any final conclusion, which can be explained by the very low incidence of cranial and spinal chordoma. Since a large number of the investigated tumor materials had routinely been embedded in paraffin over a longer period of time, strict RNase-free treatment could only be guaranteed from the time of the examinations. Therefore, possible "false negative results" in in situ hybridization are conceivable. We did not subject the biopsies to different decalcifying time periods, but overnight decalcification similar to the situation in the daily clinical practice. Nevertheless, differences in outcome between studies are probably caused by different decalcifying periods, so it would be worthwhile to further elucidate this aspect.

To avoid a technical pitfall that might lead to false-negative results, it is important that the signal be at least as strong as the control signal so that control gene expression signifies that enough RNA is preserved to allow detection of any positive cells. Furthermore, by taking biopsies of the tumor to enable different decalcification conditions, we probably introduced some heterogeneity in samples. We are aware of this side issue of our study design.

Systemic treatments of chordoma are largely ineffective and new therapeutic approaches are therefore needed. Recently, survivin expression has been suggested for use as a potential target gene of angiogenesis in sacral chordoma [66]. We include only conventional (classic) cranial and spinal chordoma and their recurrences in this study. We assume that the HH signal cascade may play an important role in conventional cranial and spinal chordoma and their recurrences. Due to the high Shh and GLI1 expression levels in all investigated chordoma samples, the study suggests a possible autocrine ligand-dependent activation of the canonical HH signaling cascade. A non-canonical, but also a paracrine mechanism is also conceivable based on our findings, which may be parallel, leading to tumor progression.

Chemotherapy with HH inhibitors - in the sense of Shh, GLI- and SMO-inhibitors - could represent a therapeutic approach in many chordoma patients with multiple relapses. The implementation of a multicenter study would therefore be valuable.

## Figures and Tables

**Table 1:** Overview of the patient collective (*n* = 20).

	Cranial Chordoma	Spinal Chordoma
Number of patients (*n* = 20)	12 (60%)	8 (40%)
• Female	6 (50%)	5 (62%)
• Male	6 (50%)	3 (38%)
1. Recurrence	5 (42%)	5 (63%)
• Female	4 (80%)	2 (40%)
• Male	1 (20%)	3 (60%)
2. Recurrence	2 (17%)	0
• Female	1 (50%)	0
• Male	1 (50%)	0
Average age	49 Y.	57 Y.
Age range	10 Y.–89 Y.	18 Y.–80 Y.

**Table 2:** Immunohistochemistry: distribution and responsiveness of the cranial chordomas in eight patients and 14 samples divided into seven primary tumors, five first relapses and two second recurrences.

Cranial Chordoma	Shh (+) Primary Tumors (*n* = 7)	Gli1 (+) Primary Tumors (*n* = 7)	Shh (+) 1.Recurrences (*n* = 5)	Gli1 (+) 1.Recurrences (*n* = 5)	Shh (+) 2.Recurrences (*n* = 2)	Gli1 (+) 2.Recurrences (*n* = 2)
Score 0	0 (0%)	0 (0%)	2 (40%)	0 (0%)	2 (100%)	0 (0%)
Score 1	3 (43%)	2 (29%)	0 (0%)	0 (0%)	0 (0%)	1 (50%)
Score 2	1 (14%)	1 (14%)	3 (60%)	2 (40%)	0 (0%)	1 (50%)
Score 3	3 (43%)	4 (57%)	0 (0%)	3 (60%)	0 (0%)	0 (0%)

**Table 3:** Immunohistochemistry: Distribution and reaction strength of spinal chordomas in six patients and 18 samples divided into twelve primary tumors and six first recurrences.

Cranial Chordoma	Shh (+) Primary Tumors	Gli1 (+) Primary Tumors	Shh (+) 1.Recurrences	Gli1 (+) 1.Recurrences
Score 0	0 (0%)	1 (8%)	0 (0%)	0 (0%)
Score 1	5 (42%)	5 (42%)	2 (33%)	1 (17%)
Score 2	5 (42%)	5 (42%)	1 (17%)	2 (33%)
Score 3	2 (16%)	1 (8%)	3 (50%)	3 (50%)

**Table 4:** In situ hybridization: response of the cranial chordomas in 8 patients corresponding 14 samples that divided into seven primary tumors, 5 first relapses and two-second recurrences.

Chordoma	Primary Tumors *n* = 7	1.Recurrences	2.Recurrences
Cranial chordoma			
Ptch1 (+)	2 (29%)	0 (0%)	0 (0%)
Gli1 (+)	2 (29%)	0 (0%)	0 (0%)
Spinal chordoma			
Ptch1 (+)	3 (25%)	0 (0%)	0 (0%)
Gli1 (+)	5 (42%)	0 (0%)	0 (0%)

## Abbreviations

HH	Hedgehog
SHH	sonic hedgehog
GLI1	glima-associated oncogene 1
SMO	Smoothened,
DHH	Desert Hedgehog
PTCH	Patched 1 protein
RAS	Rat Sarcoma
TGFb	Transforming Growth Factor Beta.
